# Diagnostic value of circulating tumor cell detection in bladder and urothelial cancer: systematic review and meta-analysis

**DOI:** 10.1186/1471-2407-11-336

**Published:** 2011-08-04

**Authors:** Pavlos Msaouel, Michael Koutsilieris

**Affiliations:** 1 Department of Experimental Physiology, Medical School, National and Kapodistrian University of Athens, 75 Micras Asias str., Goudi-Athens 115 27, Greece; 2 Department of Internal Medicine, Jacobi Medical Center, Albert Einstein College of Medicine, Bronx, NY, USA

## Abstract

**Background:**

The diagnostic value and prognostic significance of circulating tumor cell (CTC) detection in patients with bladder cancer is controversial. We performed a meta-analysis to consolidate current evidence regarding the use of CTC detection assays to diagnose bladder and other urothelial cancers and the association of CTC positivity with advanced, remote disease.

**Methods:**

Studies that investigated the presence of CTCs in the peripheral blood of patients with bladder cancer and/or urothelial cancer were identified and reviewed. Sensitivities, specificities, and positive (LR+) and negative likelihood ratios (LR-) of CTC detection in individual studies were calculated and meta-analyzed by random effects model. Overall odds ratio of CTC positivity in patients with advanced disease versus those with organ-confined cancer was also calculated.

**Results:**

Overall sensitivity of CTC detection assays was 35.1% (95%CI, 32.4-38%); specificity, LR+, and LR- was 89.4% (95%CI, 87.2-91.3%), 3.77 (95%CI, 1.95-7.30) and 0.72 (95%CI, 0.64-0.81). CTC-positive patients were significantly more likely to have advanced (stage III-IV) disease compared with CTC-negative patients (OR, 5.05; 95%CI, 2.49-10.26).

**Conclusions:**

CTC evaluation can confirm tumor diagnosis and identify patients with advanced bladder cancer. However, due to the low overall sensitivity, CTC detection assays should not be used as initial screening tests.

## Background

Bladder cancer is an important cause of morbidity and mortality with an estimated 386,300 new cases and 150,200 deaths occurring worldwide in 2008 alone [1]. The highest bladder cancer incidence rates are reported in Europe, North America, and Northern Africa and the majority of cases occur in men [1,2]. Urothelial (transitional cell) carcinomas are the most common histological type of bladder cancer. Non-urothelial vesical tumors are extremely rare and account for < 5% of all primary bladder malignancies combined [3]. Approximately 95% of primary urothelial cell cancers arise from the bladder and only a few cases originate from other sites within the urinary tract such as the renal pelvis and ureter [4-6].

The most widely used system for bladder cancer staging at this time is the American Joint Committee on Cancer (AJCC) tumor, lymph node, and hematogenous metastasis (TNM) system [7]. According to this system, extravesical disease is categorized as either stage III or IV tumors invading adjacent tissues and/or metastasizing to lymph nodes or to distant sites, whereas stage ≤II cancers are localized (organ-confined). However, initial clinical staging can be imprecise and a considerable proportion of patients thought to have localized disease will be upstaged to extravesical cancer following surgical treatment [8]. Inaccurate clinical staging may lead to suboptimal treatment, particularly since extravesical disease at the time of surgical therapy is a known predictor of poor prognosis [9,10] and patients who are thought to have localized disease may not receive potentially beneficial neoadjuvant therapy. Increased accuracy of initial clinical staging would thus facilitate risk stratification and preoperative decision making.

During the initial sequences of metastatic progression, cancer cells originating from the primary site intravasate into the lymphatics and systemic circulation as circulating tumor cells (CTCs) [11,12]. Although the majority of CTCs will either die in the bloodstream due to mechanical shear forces, immune surveillance, and/or other regulatory mechanisms, a few cells will successfully extravasate and form new colonies at distant sites. A variety of methods for detecting CTCs have been developed including nested RT-PCR, which utilizes two pairs of PCR primers to amplify a single locus. PCR-based methods are considered highly sensitive and also to demonstrate strong specificity via the design of primers that detect mRNA expression of tumor-specific genes such as cytokeratin (CK)-20, uroplakin (UP) II, and epidermal growth factor receptor (EGFR) [13,14]. The CellSearch system is another commonly used technique that was recently approved by the US Food and Drug Administration (FDA) for CTC detection in patients with metastatic breast, colorectal, and prostate cancer. CellSearch is a semi-automated, standardized, enrichment and detection system that uses magnetically labeled anti-EpCAM antibodies to capture CTCs that are then visualized and enumerated by digital fluorescent microscopy [15].

The presence of CTCs in the circulation may signify an early step of the metastatic process, which may be followed by establishment of clinically undetectable micrometastatic foci that will ultimately grow into clinically apparent metastasis. However, clinical reports evaluating molecular detection of CTCs have given contradictory and inconclusive results with some studies indicating that CTC detection may be associated with higher-stage disease [16-22] whereas others failed to show such an association [23-27]. We used meta-analytic approaches to pool together and summarize quantitatively the available evidence with regards to diagnostic accuracy of CTC detection in bladder and urothelial cancer patients as well as clarify whether detection of these cells is associated with higher stage, non-organ-confined disease.

## Methods

### Publication Search

We conducted a computerized search in April 2011 (last search, April 18 2011) without time restrictions using the electronic databases PubMed, SciVerse Scopus, Google Scholar, and the World Health Organization (WHO) International Clinical Trials Registry Platform. The search strategy included the following keywords variably combined: "bladder cancer," "circulating tumor cells," "circulating urothelial cells," "circulating bladder cancer cells," "minimal residual disease," "peripheral blood," "serum," "polymerase chain reaction," "immunomagnetic cell enrichment," "CellSearch," "CK19/CK20/uroplakin/EGFR/survivin mRNA," "micrometastasis," "urothelial cancer," "transitional cell cancer," "molecular staging," and "bladder cancer cell enrichment." We evaluated all associated publications to retrieve the most eligible studies. Moreover, their reference lists were searched manually to find other relevant publications. Both original and review articles were sought because the latter were considered an additional source of unaccounted original works.

### Inclusion and Exclusion Criteria

Eligibility criteria for further meta-analysis of the studies included: 1) publication in a peer-reviewed journal; 2) primary cohort of patients with bladder cancer and/or urothelial cancer originating from other locations (animal models or *in vitro *cell line studies were excluded); and 3) clearly identified negative controls (healthy volunteers, nonmalignant bladder disease patients, or patients with prior history of urothelial cancer but no evidence of recurrence) and/or sufficient data to extrapolate the AJCC stage of the patients. Major exclusion criteria were the number of patients enrolled--there had to be ≥20 patients or ≥30 patients and controls for a study to be considered eligible--and duplication of results from a previous publication. Duplicate populations were included in the analysis only if they were investigated with multiple molecular methods and/or tumor markers. Data from letters to the editor, meetings abstracts, preliminary reports, and non-English language papers were not considered eligible.

### Data Extraction

The two investigators independently reviewed and extracted the following data from all eligible publications according to the inclusion and exclusion criteria: first author's surname, year of publication, country of origin, study population characteristics (including no. patients enrolled, age, source of the control groups and cancer stages with distribution of patients among stages), outcomes measured (diagnostic accuracy, correlation with stage, recurrence-free survival, and overall survival), sampling site (peripheral blood, bone marrow, lymph nodes, tumor tissue biopsies), timing of blood withdrawal (preoperative, intraoperative, postoperative, before, during, or after chemotherapy), blood sample volume, no. blood samples per patient, method of CTC isolation and enrichment, molecular technique, target gene, and/or antigen used for CTC detection, *in vitro *sensitivity of each molecular method (if assessed), and no. subjects found CTC positive (CTC+) or negative (CTC-) using each molecular method. In cases where multiple blood samples were collected, CTC status of the preoperative (pretreatment) sample was used in the analysis. When more than one pretreatment blood sample was analyzed per patient, we considered as CTC+ those cases where at least one pretreatment sample/analysis was positive. Disagreements were resolved by iteration, discussion, and consensus between the two authors.

### Statistical Analysis

Statistical pooling of diagnostic accuracy variables (sensitivity, specificity, likelihood ratios) was performed using Meta-DiSc software (Version 1.4) [28]. Potential variations due to threshold effect were assessed graphically by visual inspection of accuracy estimates pairs in forest plots and sROC curves as well as statistically by computing the Spearman correlation coefficient between the logit of sensitivity and the logit of 1 - specificity [28,29]. The positive likelihood ratio (LR+) was defined as percent bladder cancer patients (and/or patients with urothelial cancer in other locations within the urinary tract) who had positive CTC detection (CTC+) divided by that of control subjects who were CTC+. The negative likelihood ratio (LR-) was defined as percent bladder and urothelial cancer patients with CTC-negative results (CTC-) divided by that of control subjects who were CTC-. To assess between-study heterogeneity (other than threshold effect) and between-study inconsistency Cochran Q statistic and inconsistency index (I^2^) were calculated and the level of significance for the corresponding P-value was set at P = 0.10. Due to anticipated interstudy heterogeneity, a random effects analysis model (DerSimonian Laird) [30] was applied in all meta-analytic calculations performed because it provides more conservative estimates of the pooled data. Pooling of individual studies to calculate odds ratios (OR) of CTC positivity in patients with extra-organ and/or lymph node-positive and metastatic disease (stage III-IV patients) compared with those with organ-confined cancer (stage ≤ II) was performed using the MIX 2.0 Pro software (version 2.0.1.2) [31]. Publication bias was assessed by visual inspection of the funnel plot [32] and statistically using Egger's linear regression test [33]. To be more conservative the level of statistical significance for the interpretation of Egger's test was set at P = 0.10. In cases of cells containing zero values for no. events of interest, continuity correction was implemented by addition of 0.5. To assess stability of the meta-analyses' results one-way sensitivity analysis was performed by omitting each study (one at a time) from the meta-analysis. In cases were studies evaluated multiple markers independently we combined the data of each assay separately following the approach used in a recent meta-analysis of CTCs in breast cancer [34]. Because this strategy may compromise interstudy independence, sensitivity analyses to assess its statistical effect on our model were performed by including the results of a single marker for each study using either the marker with the best specificity or the best sensitivity (in cases were specificity was equal between markers). To evaluate the effects of potential sources of heterogeneity in the pooled calculations subgroup analysis was performed considering more homogenous set of studies that adopted similar design variables. Subgroups were constructed only when ≥3 studies could be included. Tests of interaction were performed to assess differences between subgroups [35]. Values of 95% confidence intervals (CI) were used for all pooled data; all P-values are two tailed and those < 0.05 were considered statistically significant unless otherwise specified. To correct for multiplicity of comparisons in subgroup analyses, P-values of paired comparisons between subgroups were adjusted by Bonferroni-Holm procedure [36].

## Results

### Search Results

**Figure 1:**
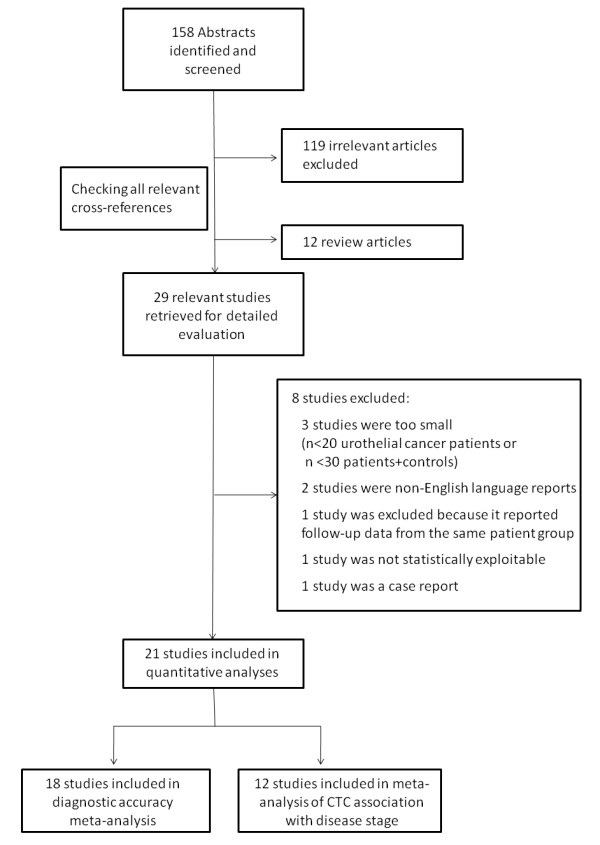
**Study flowchart of selection of eligible studies included in the meta-analyses computations**.

The study flowchart is illustrated in Figure 1. The search criteria yielded 158 abstracts of which 119 were irrelevant and 12 review articles on molecular staging of bladder cancer. Twenty-nine relevant studies were identified [16-27,37-53] and reviewed in detail. Eight of these studies did not meet the inclusion criteria: 3 studies had enrolled < 20 patients or < 30 patients and controls [17,46,47]; 2 were not written in English [52,53], 1 [51] reported follow-up data from a patient cohort originally assessed in a previous report [50]; 1 article did not include controls in the study design and all enrolled patients had stage IV urothelial cancer [44]; and 1 article was a case report of 2 patients with metastatic urothelial cancer [42]. Therefore 28 articles were included in further meta-analysis calculations: 18 articles [16,19-21,23-25,27,37-41,43,45,48-50] were included the meta-analysis of diagnostic accuracy of CTC detection (combined total of 764 patients and 708 controls) and 12 articles [16,18-20,22-26,39,41,50] were included in the meta-analysis of CTC association with disease stage (combined total of 331 patients with stage ≤II cancer and 203 with advanced-stage III-IV cancer).

### Baseline characteristics of identified studies

Patients' baseline characteristics and study design variables of the included articles are summarized in Table 1 and Additional file 1, respectively. Mean no. patients enrolled was 41 (range, 4-108) with 7 of 21 studies (33.3%) enrolling > 50 patients. Considering the 18 studies that compared CTC presence among patients and control groups, mean no. control subjects was 39 (range 2-344). Tumor histology was reported in 14 of 21 studies (66.7%). All patients in these 14 studies were diagnosed with transitional cell carcinoma with 2 patients in one study [19] showing additional squamous and adenocarcinoma components, respectively. Cancer originated from the bladder in 829 of 869 cases (95.4%), bladder and ureter in 1 of 869 cases (0.12%), upper urinary tract (renal pelvis and ureter) in 38 of 869 cases (4.4%), and urethra in 1 of 869 cases (0.12%).

**Table 1:** Baseline characteristics of patients in the 21 eligible studies included in the meta-analyses

First author (year of publication) (reference)	Country of origin	CTC+ patients (marker used)	CTC+ controls (marker used)	Patient age (years)		Tumor histology	Tumor location	Tumor stage (AJCC)	Rate of CTC+ stage ≤II pts (marker used)	Rate of CTC+ stage III-IV pts (Marker used)
									
				Median (range)	Mean (range)					
Gazzaniga (2005)[37]	Italy	11/19 (Tenascin C), 11/19 (EGFR)	0/40 (Tenascin C) 0/40 (EGFR)	NR	NR	NR	B	I-IV	NR	NR
Ribal (2006)[23]	Spain	23/70	0/22	65 (44-81)	-	TCC	B	0a-IV	4/31	17/39
Champelovier (1999)[38]	France	3/4	21/29	NR	NR	NR	B	NR	NR	NR
Okegawa (2004)[39]	Japan	25/108 (UP II), 31/108 (CK-20)	Healthy volunteers: 0/20 for either marker; nonmalignant bladder disease patients: 0/10 for UP II and 2/10 for CK-20	Bladder cancer and nonmalignant bladder patients: 57 (42-75); healthy volunteers: 41 (21-52)	-	TCC	B	0a-IV	20/91 (CK-20) 14/91 (UP II)	11/17 (CK-20) 11/17 (UP II)
Retz (2001)[24]	Germany	2/20	0/10 (isolated PBMN)	(34-79)	-	NR	B	0a-IV	0/14	2/6
Gudemann (2000)[16]	Germany	12/49	Healthy volunteers: 0/22; urocystitis patients: 0/6; benign renal tumor patients: 0/6; patients with prior history of urothelial cancer but no evidence of recurrence: 0/4	Cancer patients: 68 (60-75); urocystitis patients: 72 (68-74)	-	TCC	B: 48/49 U/P: 1/49	0a-IV	5/35	7/14
Li (1999)[40]	U.S.A.	3/60	0/10	NR	NR	TCC	B	NR	NR	NR
Soria (2002)[25]	France	27/30	0/17	Patients: 68.5 (49-99); controls: (26-58)	-	NR	B	0-IV	14/15	13/15
Desgrandchamps (1999)[41]	U.K.	1/31	0/2 (initially thought to have malignant bladder disease, one patient was found to have nonspecific inflammation and one schistosomiasis)	NR	NR	TCC	B	0a-IV	0/25	1/6
Naoe (2007)[18]	Japan	8/26	No controls assessed	70.5 (55-85)	-	TCC	B: 22/26 U: 3/26 P: 1/26	0a-IV	0/8	8/18
Kinjo (2004)[19]	Japan	18/38	0/18 (UTI n = 6; BPH n = 7; other benign conditions n = 5)	Cancer patients: 67 (35-87); controls:56 (18-78)	-	TCC:36/38; TCC+sq:1/38; TCC+ad:1/38	B	0a-IV	13/33	5/5
Guzzo (2009)[26]	U.S.A.	9/43	No controls assessed	-	67.5 (46-83)	TCC	B	0a-IV	2/17	7/26
Allard (2004)[43]	U.S.A.	6/7	8/145 healthy pre- and post-menopausal women had 1 CTC; 14/199 women with benign breast diseases had 1 CTC (none had ≥2 CTCs)	NR	NR	NR	B	IV	NR	NR
Lu (2000)[20]	Japan	15/56	0/10 healthy volunteers; 0/10 patients with renal cell cancer	71.5 (35-87)	69.41	TCC	B: 42/56 B/U: 1/56 U: 9/56 P: 4/56	0a-IV	4/34	11/22
Osman (2004)[45]	U.S.A.	21/48 (UP Ia), 10/48 (UP Ib), 25/48 (UP II), 11/48 (UP III), 26/48 (EGFR)^a^	5/14 (UP Ia), 7/14 (UP Ib), 5/14 (UP II), 10/14 (UP III), 10/14 (EGFR)^a^	64 (42-88)	-	NR	B	III-IV	NR	NR
Rink (2011)[27]	Germany	20/55	1/10	Patients: 67 (44-89); healthy volunteers: 45	Patients:66; healthy volunteers: 46	TCC	B	0a-IV	NR	NR
Fujii (1999)[21]	Japan	9/40	0/25	NR	NR	TCC	B: 27/40 U/P: 12/40 Urethra: 1/40	0a-IV	NR	NR
Okegawa (2010)[22]	Japan	11/36	No controls assessed	Stages I-II patients: 71; stage III-IV patients: 68	-	TCC	B: 28/36 U/P: 8/36	I-IV	0/16	11/20
Gradilone (2010)[48]	Italy	24/54 (CD45-/CK8+), 22/54 (survivin)	0/20	57.5 (51-64)	-	NR	B	I	NR	NR
Meye (2002)	Germany	18/34	0/20	NR	NR	TCC	B	NR	NR	NR
Gazzaniga (2001)[50]	Italy	20/27 (EGFR); 17/27 (UP II); 11/27 (CK-19); 4/27 (CK-20)	Healthy volunteers: 0/30 (EGFR), 0/30 (UP II), 6/30 (CK-19), 4/30 (CK-20); cystitis patients: 0/9 (EGFR), 3/9 (UP II), 3/9 (CK-19), 2/9 (CK-20)	NR	NR	TCC	B	Oa-IV	2/8 (EGFR) 7/8 (UP II) 1/8 (CK-19) 1/8 (CK-20)	18/19 (EGFR) 3/19 (UP II) 10/19 (CK-19) 3/19 (CK-20)

^a^Bladder cancer patients in this study were defined as those with disease at the end of follow up; controls were defined as those without disease at end of follow up

B, bladder; BPH, benign prostatic hyperplasia; CK, cytokeratin; CTC+, circulating tumor cell positive; EGFR, epidermal growth factor receptor; NR, not reported/retrievable; P, renal pelvis; PBMN, peripheral blood mononucleocytes; pts, patients; TCC, transitional cell cancer; TCC+ad, transitional cell cancer with adenocarcinoma component; TCC+sq, transitional cell cancer with squamous component; U, ureter; UP, uroplakin; UTI, urinary tract infection.

Peripheral blood samples were collected before any treatment in 11 of 21 studies (52.4%), before therapy or ≥7 days postchemotherapy in 2 of 21 studies (9.5%) whereas 8 of 21 studies (38.1%) used miscellany sampling schedules or did not report the sample collection timing. Mean volume of analyzed blood samples was 8.6 (range, 2-16) ml with 10 of 21 (47.6%) studies using ≤7.5 ml blood for their assays. Six of 21 studies (28.6%) collected two consecutive blood samples, processing only the second tube and discarding the first tube to avoid contamination by Merkel cells. With regards to CTC enrichment from peripheral blood samples 6 of 21 studies (28.6%) used Ficoll-Hypaque centrifugation, 2 studies (9.5%) included further enrichment methods in addition to Ficoll-Hypaque centrifugation, 2 studies (9.5%) used guanidinium thiocyanate-phenol-chloroform extraction, 3 studies (14.3%) adopted red blood cell lysis protocols, 1 study (4.8%) used succinyl-linked gelatin separation, 5 studies (23.8%) used the CellSearch system to enrich and detect CTCs, and 1 study (4.8%) applied a different immunomagnetic technique. One study (4.8%) did not report a cell enrichment method.

CTC detection was based on PCR in 13 of 21 studies (61.9%), of which nested RT-PCR was the most frequently used (9 of 13 studies) whereas single-round PCR was used in 4 studies, two of which were enhanced with either Southern blot analysis (1 study) or immunobead isolation (1 study). Five of 21 studies (23.8%) used the CellSearch system for CTC detection, 2 studies (9.5%) used immunocytochemistry-based methods, and 1 study (4.8%) used an ELISA-based telomerase assay. Among the PCR-based methods, 8 of 13 studies (61.5%) evaluated 1 marker using RT-PCR-based techniques whereas 4 of 13 (30.8%) studies assessed mRNA expression of ≥2 different markers in the same patient and control populations. Only two of these studies [45,50] additionally provided data on multiple marker combination in bladder cancer patients whereas the other two studies [37,39] reported the presence of each marker individually. One study [48] defined CTCs as cells lacking CD45 mRNA but expressing CD8 mRNA (CD45^-^/CD8^+ ^by PCR detection) and we maintained this definition when analyzing data from that report. The most commonly used marker for PCR-based techniques was CK-20 (evaluated in 7 of 13 studies; 53.8%) followed by UP II (5 of 13 studies; 38.4%) and EGFR (3 of 13 studies; 23.1%) whereas tenascin C, MUC7, UP Ia, UP Ib, UP III, and CK-19 were evaluated in 1 of 13 (7.7%) studies each.

*In vitro *sensitivity of circulating bladder/urothelial cancer cell detection methods was reported in 11 of 21 studies (52.4%) (Additional file 1). In 9 of 11 studies (81.8%) it was determined by spiking experiments that 1-10 bladder cancer cells could be detected in 10^6 ^normal cells (or cells not expressing the tumor marker) or in 5 ml blood. Two of 11 studies (18.2%) used the CellSearch system and reported a mean recovery rate of 85.5% (range, 78.5-94%) in 7.5-10 ml blood.

### CTC Diagnostic Value

**Figure 2:**
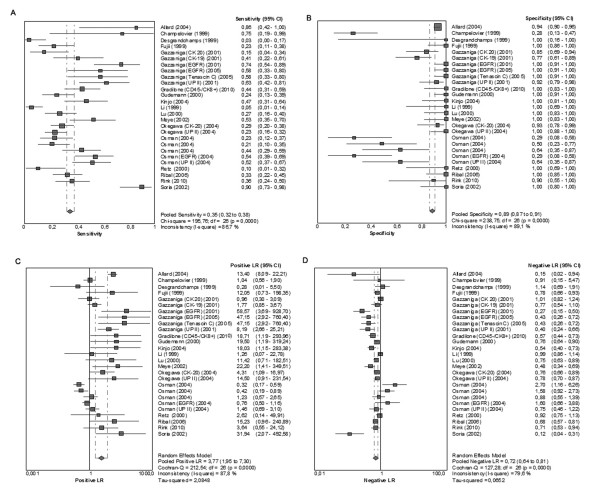
**Diagnostic accuracy forest plots**. Forest plots of the overall sensitivity **(A)**, specificity **(B)**, positive likelihood ratio (LR+) **(C)**, and negative likelihood ratio (LR-) **(D) **of circulating tumor cell detection are presented. The size of each square is proportional to sample size. Horizontal lines in each square show the corresponding 95% confidence intervals (CI). The center of the diamond indicates the overall sensitivity, specificity, LR+, and LR- and the ends correspond to 95%CI.

When all eligible studies and assays were pooled into the diagnostic accuracy meta-analysis, the overall sensitivity of CTC detection was 35.1% (95%CI, 32.4-38%; I^2 ^= 86.7%; Figure 2A); overall specificity LR+, and LR- was 89.4% (95%CI, 87.2-91.3%; I^2 ^= 89.1%; Figure 2B), 3.77 (95%CI, 1.95-7.30; I^2 ^= 87.8%; Figure 2C), and 0.72 (95%CI, 0.64-0.81; I^2 ^= 79.6%; Figure 2D). The included studies were significantly heterogenous in their estimates of sensitivity, specificity, LR+, and LR- (all Q statistic P < 0.001; all I^2 ^> 75%). Threshold effect was not detected (Spearman r = -0.115; P = 0.567). The stability of our model was confirmed by leave-one-out sensitivity analysis, which generated pooled estimates very close to those obtained with all eligible studies (mean sensitivity [range], 35.2%, 33.7-36.8%; mean specificity [range], 89.4%, 87-91.3%; mean LR+ [range], 3.79, 3.44-4.25; mean LR- [range] 0.72, 0.70-0.74). Single-marker sensitivity analysis of the 18 studies, estimated by using only the most specific (or most sensitive in cases with equal specificity) assay to define CTC positivity in each study, showed a pooled sensitivity 34.9% (95%CI, 31.5-38.5%; I^2 ^= 89.3%), specificity 93.1% (95%CI, 91.0-94.8%; I^2 ^= 87.3%), LR+ 7.17 (95%CI, 3.032-16.943; I^2 ^= 78.4%;), and LR-0.66 (95%CI, 0.56-0.78; I^2 ^= 85.9%;).

The results of subgroup analyses are summarized in Table 2. Studies conducted in Italy showed significantly higher sensitivity compared with those conducted in Germany (adjusted P = 0.0099), Japan (adjusted P < 0.001), and the USA (adjusted P = 0.0033). Studies conducted in the USA also showed significantly lower specificity compared with those conducted in Italy or Japan (adjusted P < 0.001). Studies conducted in countries other than Germany, Italy, Japan, or the USA showed significantly lower specificity compared with those originating from Germany (adjusted P < 0.001), Italy (adjusted P < 0.001), Japan (adjusted P < 0.001), or the USA (adjusted P = 0.0015). Studies that did not report the histological type of the cancer had significantly higher sensitivity and specificity compared with those that reported this parameter (adjusted P < 0.001). Furthermore, studies in which blood samples were collected either before treatment or ≥7 days after the last chemotherapy cycle demonstrated significantly lower sensitivity (adjusted P = 0.025) but higher specificity (adjusted P < 0.001) compared with those with other timeframes.

**Table 2:** Subgroup analyses of diagnostic accuracy variables

Parameter	**Subgroups**^**a**^	**Sensitivity**^**b**^	**Specificity**^**b**^	**Positive likelihood ratio**^**b**^	**Negative likelihood ratio**^**b**^
Country of origin	Germany (n = 4)	32.9% (25.7 to 40.8%; I^2 ^= 77.8%)	98.7% (93.1 to 100%; I^2 ^= 28.6%)	6.86 (1.99 to 23.63; I^2 ^= 0)	0.72 (0.57 to 0.92; I^2 ^= 75.1%)
	Italy (n = 3)	55.0% (44.7 to 65.0%; I^2 ^= 70.0%)	100% (96.3 to 100%; I^2 ^= 0)	37.15 (7.53 to 183.47; I^2 ^= 0)	0.43 (0.26 to 0.71; I^2 ^= 74.7%)
	Japan (n = 4)	27.7% (22.1 to 33.8%; I^2 ^= 64.1%)	100% (96.1 to 100%; I^2 ^= 0)	13.79 (3.45 to 55.22; I^2 ^= 0)	0.74 (0.65 to 0.83; I^2 ^= 47.8%)
	USA (n = 3)	29.6% (21.4 to 38.8%; I^2 ^= 95.4%)	92.7% (89.5 to 95.1%; I^2 ^= 82.2%)	3.25 (0.27 to 38.72; I^2 ^= 95.4%)	0.60 (0.18 to 1.97; I^2 ^= 93.4%)
	Other (n = 4)	40.0% (31.7 to 48.8%; I^2 ^= 95.0%)	70.0% (57.9 to 80.4%; I^2 ^= 94.2%)	3.34 (0.11 to 104.66; I^2 ^= 90.6)	0.55 (0.22 to 1.38; I^2 ^= 89.0%)
Histologic tumor type	TCC ± other components (n = 11)	28.9% (25.2 to 32.8%; I^2 ^= 87.5%)	99.6% (97.6 to 100%; I^2 ^= 0)	8.42 (3.5 to 20.29; I^2 ^= 15.6%)	0.71 (0.61 to 0.83; I^2 ^= 82.1%)
	Not reported (n = 7)	53.8% (46.3 to 61.2%; I^2 ^= 85.7%)	89.9% (86.8 to 92.4%; I^2 ^= 93.7%)	5.89 (1.61 to 21.62; I^2 ^= 89.2%)	0.49 (0.28 to 0.85; I^2 ^= 89.2%)
Sampling time	Pretreatment or ≥7 days post-chemotherapy (n = 11)	31.0% (27.0 to 35.2%; I^2 ^= 50.0%)	95.8% (93.8 to 97.4%; I^2 ^= 53.1%)	11.57 (7.28 to 18.40; I^2 ^= 1.3%)	0.71 (0.62 to 0.81; I^2 ^= 71.2%)
	NR or miscellany (n = 7)	43.2% (36.9 to 49.7%; I^2 ^= 93.8%)	83.1% (76.2 to 88.7%; I^2 ^= 93.1%)	5.69 (1.16 to 27.97; I^2 ^= 86.7%)	0.52 (0.30 to 0.89; I^2 ^= 94.2%)
Blood sample volume	≤7.5 ml (n = 8)	31.3% (26.6 to 36.4%; I^2 ^= 89.2%)	88.8% (83.5 to 92.8%; I^2 ^= 92.8%)	8.16 (1.17 to 56.83; I^2 ^= 85.8%)	0.64 (0.49 to 0.85; I^2 ^= 89.7%)
	> 7.5 ml (n = 10)	38.4% (33.5 to 43.5%; I^2 ^= 89.9%)	94.7% (92.4 to 96.5%; I^2 ^= 69.6%)	7.15 (2.52 to 20.32; I^2 ^= 71.2%)	0.67 (0.54 to 0.83; I^2 ^= 82.4%)
Collection of two consecutive blood samples	Yes (n = 6)	41.2% (35.2 to 47.5%; I^2 ^= 90.3%)	100% (97.1 to 100%; I^2 ^= 0)	15.32 (4.91 to 47.84; I^2 ^= 0)	0.61 (0.46 to 0.81; I^2 ^= 87.6%)
	No (n = 12)	31.6% (27.6 to 36.0%; I^2 ^= 89.0%)	91.6% (89.0 to 93.7%; I^2 ^= 90.3%)	5.33 (1.93 to 14.68; I^2 ^= 83.0%)	0.69 (0.56 to 0.85; I^2 ^= 85.4%)
Cell separation method	Ficoll-Hypaque centrifugation ± further methods (n = 8)	34.4% (29.2 to 39.9%; I^2 ^= 90.5%)	87.9% (82.0 to 92.3%; I^2 ^= 92.5%)	6.99 (0.88 to 55.73; I^2 ^= 85.9%)	0.69 (0.56 to 0.84; I^2 ^= 76.8%)
	RBC lysis protocols (n = 3)	35.1% (28.4 to 42.2%; I^2 ^= 87.0%)	91.9% (82.2 to 97.3%; I^2 ^= 87.9%)	5.74 (0.50 to 65.75; I^2 ^= 75.8%)	0.69 (0.52 to 0.91; I^2 ^= 66.4%)
	Other protocols or NR (n = 7)	35.5% (29.5 to 41.9%; I^2 ^= 91.3%)	95.1% (92.8 to 96.9%; I^2 ^= 56.8%)	10.77 (4.92 to 23.56; I^2 ^= 19.9%)	0.57 (0.36 to 0.92; I^2 ^= 94.3%)
Molecular detection technique	RT-PCR based (including nested RT-PCR; n = 13)	32.0% (28.3 to 36.0%; I^2 ^= 85.8%)	91.7% (88.1 to 94.5%; I^2 ^= 90.6%)	7.75 (2.38 to 25.24; I^2 ^= 80.2%)	0.70 (0.61 to 0.81; I^2 ^= 81.8%)
	Other (n = 5)	45.9% (37.9 to 54.0%; I^2 ^= 93.7%)	94.1% (91.3 to 96.3%; I^2 ^= 22.2%)	7.60 (2.17 to 26.67; I^2 ^= 53.3%)	0.45 (0.22 to 0.94; I^2 ^= 89.4%)
Nested RT-PCR method	Yes (n = 9)	30.5% (26.2 to 35.1%; I^2 ^= 71.6%)	87.4% (82.1 to 91.6%; I^2 ^= 92.3%)	5.37 (1.58 to 18.27; I^2 ^= 77.7%)	0.76 (0.70 to 0.82; I^2 ^= 24.7%)
	No (n = 9)	41.0% (35.5 to 46.6%; I^2 ^= 93.4%)	95.4% (93.2 to 97.1%; I^2 ^= 52.1%)	10.08 (3.90 to 26.08; I^2 ^= 43.1%)	0.49 (0.29 to 0.81; I^2 ^= 94.2%)
PCR marker used^c^	CK-20 (n = 7)	26.4% (21.7 to 31.6%; I^2 ^= 48.8%)	85.0% (79.1 to 89.7%; I^2 ^= 92.6%)	3.38 (0.99 to 11.59; I^2 ^= 74.5%)	0.80 (0.73 to 0.89; I^2 ^= 43.1%)
	UP II (n = 5)	28.4% (23.4 to 33.9%; I^2 ^= 91.9%)	92.9% (86.5 to 96.9%; I^2 ^= 78.2%)	4.15 (1.20 to 14.33; I^2 ^= 62.8%)	0.75 (0.59 to 0.95; I^2 ^= 85.2%)
	EGFR (n = 3)	60.6% (50.0 to 70.6%; I^2 ^= 34.4%)	89.2% (81.1 to 94.7%; I^2 ^= 95.7%)	11.94 (0.03 to 4369.8; I^2 ^= 96.2%)	0.54 (0.23 to 1.28; I^2 ^= 81.1%)
	Other (n = 6)	36.0% (29.7 to 42.6%; I^2 ^= 69.4%)	77.7% (69.9 to 84.3%; I^2 ^= 90.2%)	1.48 (0.47 to 4.65; I^2 ^= 88.0%)	0.87 (0.56 to 1.35; I^2 ^= 81.7%)

^a^Pooled analysis performed by including results of single marker (either the most specific or the most sensitive in cases with equal specificity) in cases where multiple markers were assessed per study.

^b^Data in parentheses are 95% confidence intervals.

^c^Subgroup analyses of the different tumor markers used in PCR-based methods included and compared data from all markers in those studies where multiple assays were used.

NR, not reported/retrievable; RBC, red blood cells; TCC, transitional cell cancer.

Higher blood sample volume was significantly associated with increased sensitivity (adjusted P = 0.03) and specificity (adjusted P < 0.001). Collection of two consecutive blood samples and processing of the second tube only so as to avoid Merkel cell contamination demonstrated significantly higher specificity (adjusted P < 0.001) and no significant difference of sensitivity (adjusted P = 0.127) versus single blood sample collection. No significant difference of diagnostic accuracy variables was detected between cell separation method subgroups. RT-PCR-based techniques demonstrated significantly lower sensitivity (adjusted P = 0.03) and no significant difference of specificity (adjusted P > 0.2) compared with other molecular detection methods. Nested RT-PCR also showed significantly lower sensitivity (adjusted P = 0.046) and specificity (adjusted P = 0.0022) compared with other PCR-based and non-PCR-based CTC detection techniques. PCR-based tests assessing expression of EGFR yielded significantly higher overall sensitivity compared with CK-20 (adjusted P < 0.001), UP II (adjusted P < 0.001), and all other markers included in the meta-analysis (adjusted P < 0.001). In addition, UP II showed significantly higher overall specificity (adjusted P = 0.0048) compared with the other markers (except EGFR and CK-20). No significant difference of LR+ and LR- was detected in tests of interaction between the subgroups (all adjusted P > 0.05).

### Association of CTC detection with disease stage

**Figure 3:**
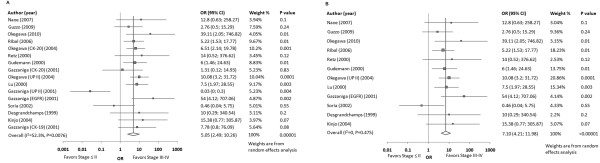
**Forest plots of association of circulating tumor cell (CTC) detection with disease stage**. Forest plots of overall association of CTC detection with disease stage were calculated by pooling data from all assays in eligible studies **(A) **and by pooling data from a single detection assay per study **(B)**. The size of each square is proportional to sample size. The center of each square and the horizontal line show the odds ratio (OR) and corresponding 95% confidence intervals (CI), respectively. The center of the diamond indicates overall OR and the ends correspond to 95%CI.

**Figure 4:**
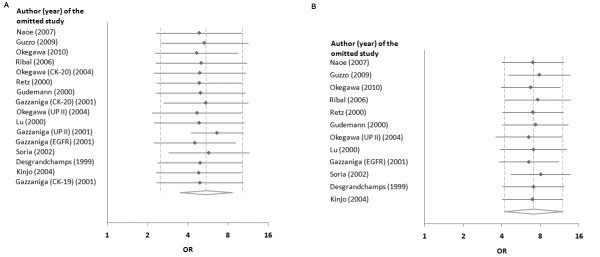
**Sensitivity analyses**. One-way sensitivity analysis of pooled data from all assays in the 12 eligible studies (A) and from a single marker assay from each of the 12 eligible studies (B). Each rectangle represents pooled odds ratio (OR); horizontal lines show corresponding 95% confidence intervals (CI) after omitting each study.

Statistical pooling of all eligible studies and assays demonstrated that CTC+ patients were significantly more likely to have advanced (stage III-IV) disease compared with CTC- patients (OR, 5.05; 95%CI, 2.49-10.26). ORs and 95%CIs from individual studies as well as the pooled calculations are shown in Figure 3A. Moderate heterogeneity among the studies was detected (Q statistic P = 0.0076; I^2 ^= 52.3%). No significant publication bias was detected as suggested by funnel plot inspection and Egger's test (intercept = -0.372; P = 0.711). Leave-one-out sensitivity analysis confirmed that our findings were not driven by any single study (Figure 4). Robustness of the model was further confirmed by sensitivity analysis using pooled data from a single marker for each study (either the most specific or the most sensitive in cases with equal specificity) revealing an overall OR 7.10 (95%CI, 4.21-11.98; I^2 ^= 0; Figure 3B). Leave-one-out analysis in the single (prioritized) marker model confirmed the lack of a dominant study and the stability of the pooled calculations (Figure 4B). Subgroup analyses are presented in Table 3. Tests of interaction did not detect any significant differences in pooled OR estimates between the various subgroups (all adjusted P > 0.4).

**Table 3:** Subgroup analyses of association with disease stage meta-analysis

Parameter	**Subgroup**^**a**^	**OR**^**b**^	Parameter	Subgroups	**OR**^**b**^
Country of origin	Japan (n = 5)	10.48 (4.82 to 22.78; I^2 ^= 0)	Histologic tumor type	TCC (n = 9)	7.91 (4.60 to 13.61; I^2 ^= 0)
	Other (n = 7)	5.20 (2.21 to 12.25; I^2 ^= 22.7%)		Not reported or TCC+other components (n = 3)	3.94 (0.35 to 43.85; I^2 ^= 51.1%)
Cell separation method	Ficoll-Hypaque centrifugation ± further methods (n = 5)	5.07 (2.48 to 10.35; I^2 ^= 0)	Sampling time	Pretreatment or ≥7 days post-chemotherapy (n = 9	8.24 (4.63 to 14.68; I^2 ^= 0)
	CellSearch method (n = 3)	7.43 (1.52 to 36.25; I^2 ^= 22.4%)		NR or miscellany (n = 3)	3.95 (0.36 to 43.95; I^2 ^= 71.0%)
	Other protocols or NR (n = 4)	13.57 (5.27 to 34.95; I^2 ^= 0)			
Blood sample volume	≤7.5 ml (n = 6)	9.36 (4.65 to 18.86; I^2 ^= 0)	Collection of two consecutive blood samples	Yes (n = 4)	4.25 (1.56 to 11.62; I^2 ^= 21.1%)
	> 7.5 ml (n = 6)	5.00 (2.27 to 11.00; I^2 ^= 0)		No (n = 8)	9.53 (4.88 to 18.62; I^2 ^= 0)
Molecular detection technique	RT-PCR based (including nested RT-PCR; n = 7)	8.36 (4.62 to 15.13; I^2 ^= 0)	Nested RT-PCR method	Yes (n = 6)	7.53 (4.09 to 13.85; I^2 ^= 0)
	Other (n = 5)	4.55 (1.07 to 19.35; I^2 ^= 34.7%)		No (n = 6)	7.21 (1.66 to 31.28; I^2 ^= 47.0%)
PCR marker used^c^	CK-20 (n = 5)	5.47 (2.80 to 10.68; I^2 ^= 0)			
	UP II (n = 3)	1.59 (0.10 to 25.27; I^2 ^= 89.8%)			
	Other (n = 3)	17.50 (3.98 to 77.03; I^2 ^= 0)			

^a^Pooled analysis was performed by including the results of single marker (either the most specific or the most sensitive in cases with equal specificity) in cases where multiple markers were assessed per study.

^b^Data in parentheses are 95% confidence intervals.

^c^Subgroup analyses of the different tumor markers used in PCR-based methods included and compared data from all markers in those studies where multiple assays were used.

NR, not reported/retrievable; OR, odds ratio; RBC, red blood cells.

## Discussion

Despite the growing enthusiasm for the use of CTC molecular detection in bladder cancer patients [13,54] the diagnostic efficacy data of these molecular methods reported to date have been variable. The present study is the first meta-analysis to estimate pooled diagnostic accuracy characteristics of CTC detection protocols in bladder cancer as well as the first report to investigate systematically associations between CTC serum markers and disease stage in these patients. Our results suggest that bladder cancer (and other urothelial cancer) CTC detection assays have limited diagnostic sensitivity because they fail to identify approximately two thirds of patients and show moderate positive and negative diagnostic likelihood ratios. On the other hand, CTC detection demonstrated high specificity for diagnosis of bladder and other urothelial cancers. Therefore CTC detection may have limited value as first-line screening or diagnostic test but may be useful in confirming the cancer diagnosis.

It should be noted that although significant evidence for the presence of a threshold effect was not detected, significant heterogeneity was present among the studies used in the estimation of diagnostic accuracy variables. Subgroup analysis showed that studies performed in Italy yielded significantly higher overall sensitivity estimates. Furthermore, studies conducted in locations other than Germany, Italy, Japan, and the USA demonstrated significantly lower overall specificity compared with that yielded in the four countries where the majority of CTC detection investigations have been performed. These findings should be further elucidated by multicenter trials as well as standardization of the techniques via collaboration of international centers.

Interestingly, studies reporting urothelial cancer histology yielded significantly lower overall sensitivity and specificity compared those that did not report the histological type of the investigated bladder cancer. The very low prevalence of nonurothelial cancer of the urinary bladder [3] suggests that the vast majority of the bladder malignancies assessed in the latter studies were of urothelial cell origin. However, further trials will be required to investigate the possibility that CTCs from primary nonurothelial bladder tumor may be more accurately detected compared with urothelial cancer using current CTC detection protocols.

An important aspect in CTC detection is the timing of the assessment because it has been suggested that surgical interventions may cause transient dissemination of CTCs in the bloodstream [55,56] whereas chemotherapy and other systemic treatments may destroy CTCs or downregulate marker expression and thus convert CTC+ patients to CTC- [57]. Our findings suggest that nontreated patients or patients who were assessed ≥7 days after the last treatment were less likely to have detectable bladder/urothelial CTCs in their bloodstream. As expected, collection of two different blood samples and discarding the first blood tube to avoid cellular contaminants such as Merkel cells deriving from skin significantly improved the specificity of CTC detection assays without affecting diagnostic sensitivity. We therefore recommend implementation of this approach particularly when RT-PCR-based techniques for detection of epithelial tumor markers are used. On the other hand, there was no detectable superiority with regards to diagnostic accuracy among the various cell enrichment techniques utilized prior to CTC detection. It should also be noted that despite the good *in vitro *sensitivity reported by the majority of studies (Additional file 1) CTC detection diagnostic accuracy was significantly higher in studies in which larger blood volumes were drawn. Taking into account the limited diagnostic sensitivity of CTC detection assays the detection limit of molecular methods should be considerably increased to allow efficient capture of CTCs in < 7.5 ml blood.

Notably, RT-PCR-based protocols yielded significantly lower sensitivity compared with other molecular detection approaches and nested RT-PCR methods in particular demonstrated significantly lower specificity. Although PCR tests do offer considerable specificity via the design of primers that are specific to the gene of interest, the specificity of PCR amplification may be compromised by a number of factors including sample contamination, illegitimate transcription (defined as low-level ubiquitous transcription of tissue-specific genes), and processed pseudogenes, which are gene sequences lacking introns that were inserted into the nuclear genome via mRNA retrotransposition [13,58]. The sensitivity of PCR assays may also be affected by several factors including primer selection, PCR conditions used, and variable expression patterns of investigated tumor markers among tumors or even CTC clones. These limitations may be addressed by the use of multimarker PCR assays. Serial testing of RT-PCR-based CTC detection protocols (whereby detection of all markers tested is required to designate a sample as CTC+) may thus improve specificity while parallel testing, considered positive if any of the markers is detected, may increase sensitivity. It is of note that only four of the PCR-based studies used in our meta-analyses calculations [37,39,45,50] assessed mRNA expression of more than one marker and only two of these studies provided data using multimarker combinations [45,50]. Furthermore, although several bladder cancer molecular markers have been identified to date [59] no single available marker (or marker combination) has been determined optimal for bladder/urothelial CTC detection. There is thus considerable heterogeneity in tumor markers used and only 3 markers (CK-20, UP II, and EGFR) have been investigated in ≥2 studies included in the present meta-analysis. We performed subgroup analyses to compare the diagnostic accuracy of different PCR-based tumor marker detection protocols and found that EGFR demonstrated the highest diagnostic sensitivity compared with all other markers tested whereas UP II yielded strong overall specificity that was significantly higher compared with all other markers other than CK-20 and EGFR. Further studies are required to corroborate these findings and to determine the optimal marker combinations for CTC detection in bladder cancer patients.

Current staging systems may inadequately guide therapeutic management of bladder cancer since many patients initially thought to have localized disease may be upstaged following operative pathology evaluation. Detection of CTCs may aid in risk stratification and treatment of bladder or other urothelial cancer patients. However, the results reported to date have been variable. The present meta-analysis showed that CTC+ bladder/urothelial cancer patients are significantly more likely to have extra-organ and/or metastatic disease; this key finding was consistently observed throughout subsequent subgroup analyses. This association indicates that CTC assessment can be used to identify patients who are more likely to be upstaged to stage III-IV cancer despite initial clinical classification into locally confined (stage ≤II) disease and who may be more likely to benefit from neoadjuvant chemotherapy. Only 4 of the 21 studies included in our meta-analyses provided data on the relation between CTC detection and progression-free survival whereas 1 of 21 studies investigated the association of CTC positivity with overall survival. Future studies should be performed to gain better knowledge in this area.

A potential limitation of the present meta-analysis is the considerable degree of interstudy heterogeneity observed. This issue was addressed by adoption of the more conservative random effects model to estimate the weights applied to each effect size as well as by following a rigorous methodological approach that excluded all studies with < 20 patients or < 30 patients and controls and required ≥3 studies per subgroup to perform pooled subgroup analyses. In addition, several subgroup and sensitivity analyses were performed to identify and address potential sources of bias. There was also some variation in the definition of healthy controls used in the studies, which may influence diagnostic accuracy results. However, the rate of false positive results was similar between different control groups such as healthy volunteers and cystitis patients in studies that investigated more than one control group (Table 1). It should also be noted that LR+ should be interpreted with caution because many studies reported zero positive CTC tests in the control groups. In such cases the denominator in the respective LR+ calculation would be zero. We addressed this issue by implementing a continuity correction of 0.5 to these control groups. However, some of the studies included only a small control population with 4 of 18 studies evaluating < 10 control subjects. In these cases the continuity correction itself may distort the LR+ calculation. In contrast, these considerations do not apply to LR- estimates.

## Conclusions

In conclusion, our results have highlighted the potential clinical role of CTC detection as an indicator of advanced bladder cancer. Our findings suggest that CTC evaluation may not be used as first-line screening test. However, the high overall specificity and consistent significant overall association with disease stage indicate the potential importance of CTC detection as a quick and noninvasive method for confirming the cancer diagnosis and as a mode of initial cancer staging. Future studies should determine the optimal tumor markers and molecular methods for CTC detection, standardize the available techniques, investigate the potential advantages of multiple marker assays in PCR-based protocols, and assess the potential correlation of CTC positivity with patient survival.

## Competing interests

The authors declare that they have no competing interests.

## Authors' contributions

PM and MK conceived the study. Data were acquired independently by both authors. PM performed data analysis and drafted the manuscript. Both authors read and approved the final manuscript.

## Pre-publication history

The pre-publication history for this paper can be accessed here:

http://www.biomedcentral.com/1471-2407/11/336/prepub

## Supplementary Material

Additional file 1 **Supplementary_Table 1**. Table S1: Study design variables of the 21 eligible studies included in the meta-analyses. Click here for file
