# Supplementary material for: Diagnostic value of circulating tumor cell detection in bladder and urothelial cancer: systematic review and meta-analysis
Source: BMC Cancer. 2011 Aug 4;11:336. doi: 10.1186/1471-2407-11-336 (PMC3161042; doi:10.1186/1471-2407-11-336)
Supplement: Additional file 1 — Supplementary_Table 1. Table S1: Study design variables of the 21 eligible studies included in the meta-analyses. [file 1471-2407-11-336-S1.DOC]

| **Supplementary Table 1. Study design variables of the 21 eligible studies included in the meta-analyses** | | | | | | | |  |
| --- | --- | --- | --- | --- | --- | --- | --- | --- |
| **First author (year of publication)**  **(reference)** | **Timing of blood withdrawal** | **Sample volume** | **No. blood samples** | **Cell separation method** | **Detection method** | **Tumor markers used** | **In vitro sensitivity** | **Additional findings/notes** |
| Gazzaniga (2005)^37^ | Pretreatment | 5 ml | Single | Guanidinium thiocyanate-phenol-chloroform extraction | RT-PCR | Tenascin C, EGFR | NR | - |
| Ribal (2006)^23^ | Pretreatment | 10 ml (second tube) | Two consecutive samples; first tube discarded to avoid contamination by Merkel cells and second sample processed | Ficoll-Hypaque centrifugation | Nested RT-PCR | CK-20 | NR | Metastatic bladder cancer "control" subjects (n = ) were included in the meta-analysis as stage IV patients |
| Champelovier (1999)^38^ | NR | 3-5 ml | Single | Ficoll-Hypaque centrifugation | Nested RT-PCR | CK-20 | 1-10 bladder cancer cells/10^6^ UM-384 leukemic cells (cell line does not express CK-20 mRNA) | - |
| Okegawa (2004)^39^ | Preop or 1 day before each chemotherapy cycle in patients with metastatic disease | 7 ml | Single (multiple in patients with metastatic disease receiving chemotherapy) | Erythrocyte lysis | Nested RT-PCR | CK-20, UP II | 1 bladder cancer cell/10^6^ normal cells | - |
| Retz (2001)^24^ | Preop (+ postop in 20 patients) | 5 ml (first tube) and 10 ml (second tube) | Two consecutive samples; first tube discarded to avoid contamination by Merkel cells and second sample processed | Guanidinium thiocyanate-phenol-chloroform extraction | Nested RT-PCR | CK-20 | 2 bladder cancer cell/10^6^ normal cells | Whole blood sampling gave false positive CK-20 mRNA |
| Gudemann (2000)^16^ | Pretreatment | 9 ml (second tube) | Two consecutive samples; first tube discarded to avoid contamination by Merkel cells and second sample processed | Ficoll-Hypaque centrifugation | Nested RT-PCR | CK-20 | NR | - |
| Li (1999)^40^ | NR for non-metastatic patients; during or before chemotherapy for metastatic patients | 5 ml | Single | NR | RT-PCR | UP II | 1 bladder cancer cell/10^6^ -10^7^ normal cells | - |
| Soria (2002)^25^ | Postop in 19 patients; NR for others | 10 ml (second tube) | Two consecutive samples; first tube discarded to avoid contamination by Merkel cells and second sample processed | Ficoll-Hypaque centrifugation + Immunomagnetic beads covalently coated with Ber-EP4 mAb (binds to epithelial cells in normal and malignant tissues) | Telomerase assay | Telomerase activity | NR | - |
| Desgrandchamps (1999)^41^ | Preop and 1-2 h Postop | 10 ml | Two (first sample preop and second sample postop) | Ficoll-Hypaque centrifugation | Immuno-cytochemistry | CK8, CK18 and CK19 | 1 bladder cancer cell/5 x 10^5^ normal cells | - |
| Naoe (2007)^18^ | Preop (n = 12), pre- or ≥1 month post-chemotherapy (n = 10) | 10 ml | Single | CellSearch | CellSearch | CellSearch | Consistent mean recovery rate 78.5 ± 5.8% HT1197 urothelial cancer cells in 10 ml blood | - |
| Kinjo (2004)^19^ | Pretreatment | 5 ml | Single | Erythrocyte lysis | Nested RT-PCR | MUC7 | 2 bladder cancer cell/1 ml blood | One patient had transitional cell + squamous cell cancer and another patient transitional cell + adenocarcinoma. |
| Guzzo (2009)^26^ | Preop (16 pts received neoadjuvant chemotherapy and 11 pts prior BCG) | 7.5 ml | Single | CellSearch | CellSearch | CellSearch | NR | In vitro sensitivity of CellSearch described extensively in previous publications^18,43^ |
| Allard (2004)^43^ | Either before or ≥7 days after IV chemotherapy | 10 ml | Single (for some patients multiple blood samples analyzed on different occasions) | CellSearch | CellSearch | CellSearch | NR for bladder/urothelial cancer cells or lines | Study enrolled 964 patients with various metastatic cancers (7 had bladder cancer) |
| Lu (2000)^20^ | Pretreatment (sequential blood sampling performed in 3 patients with nodal or distant metastasis who were treated with systemic chemotherapy) | 5 ml | Single (for 3 patients multiple blood samples analyzed on different occasions) | Ficoll-Hypaque centrifugation | Nested RT-PCR | UP II | 2 bladder cancer cells/1 ml of blood |  |
| Osman (2004)^45 a^ | Postop (n=22) patients with no evidence of disease at presentation, patients with evidence of disease (n = 40) presenting for treatment. | 8-12 ml | Single | Erythrocyte lysis | Nested RT-PCR | UP Ia; UP Ib; UP II; UP III; EGFR | NR | Patients with metastatic, locally invasive unresectable or recurrent bladder cancer (at follow-up) classified as "disease positive" (n=48); those who underwent cystectomy or had no evidence of bladder cancer after definitive surgical treatment (median follow-up, 15 months) classified as "disease negative" |
| Rink (2011)^27^ | Pretreatment | 7.5 ml | Single | CellSearch | CellSearch | CellSearch | NR | - |
| Fujii (1999)^21^ | NR | 14 ml | Single | Ficoll-Hypaque centrifugation | Nested RT-PCR | CK-20 | 10 bladder cancer cell/10^7^ normal cells | - |
| Okegawa (2010)^22^ | All patients tested for CTC before receiving treatment/chemotherapy | 7.5 ml | Single | CellSearch | CellSearch | CellSearch | Mean recovery rate 84% ± 6% for T24 urothelial cancer cells and 94% ± 5% for RT4 urothelial cancer cells in 7.5 ml blood from healthy volunteers | - |
| Gradilone (2010)^48^ | Pretreatment | 15 ml | Single (first 5 ml discarded to avoid contamination by Merkel cells) | Immunomagnetic beads covalently coated with Ber-EP4 mAb (bind to epithelial cells in normal and malignant tissues) | Immunobead PCR | CD45, CK8 (CTCs defined as CD45-/CK8+), survivin | 1 bladder cancer cell/ml blood | Study included only T1 G3 patients with no CIS, tumor size <3cm, and no multifocal tumors |
| Meye (2002) | Preop (n = 27 samples), Postop (n = 14 samples), during chemotherapy (n = 4 samples) | 16 ml | Single in some patients , multiple in others; first 5 ml discarded to minimize risk of contamination by dermal epithelial cells | Ficoll-Hypaque centrifugation + leucocyte autoMACS | Immuno-cytochemistry of Cytospin centrifugation | Pan-anti-CK antibody against CK5, CK6, CK8, CK17, and CK19 | NR for bladder/urothelial cancer cells or lines | In 5 urothelial cancer patients other malignancies were diagnosed and treated before or simultaneously to the bladder tumor diagnosis. |
| Gazzaniga (2001)^50^ | Preop (n = 19 patients with superficial or locally advanced disease), during chemotherapy (n = 1 patient with metastatic disease), 6-10 months after last chemotherapy (n = 3 patients with metastatic disease), before any treatment (n = 4 patients with metastatic disease) | 2 ml | Single | Succinyl-linked gelatin separation | RT-PCR followed by Southern blot hybridization | EGFR, UP II, CK-19, CK-20 | NR for bladder/urothelial cancer cells or lines | Follow-up results reported in a later study^51^ |
| ^a^Bladder cancer patients in this study were defined as those with disease at the end of follow up; controls were defined as those without disease at end of follow up.  autoMACS, automated immunomagnetic activated cell separation; BCG, Bacillus Calmette-Guérin; CK, cytokeratin; CTC+, circulating tumor cell-positive; EGFR, epidermal growth factor receptor; NR, not reported/retrievable; UP, uroplakin. | | | | | | | |  |
